# Omics-driven exploration and mining of key functional genes for the improvement of food and fiber crops

**DOI:** 10.3389/fpls.2023.1273859

**Published:** 2024-01-08

**Authors:** Rubab Zahra Naqvi, Muhammad Arslan Mahmood, Shahid Mansoor, Imran Amin, Muhammad Asif

**Affiliations:** ^1^ Agricultural Biotechnology Division, National Institute for Biotechnology and Genetic Engineering College Pakistan Institute of Engineering and Applied Sciences, Faisalabad, Pakistan; ^2^ International Center for Chemical and Biological Sciences, University of Karachi, Karachi, Pakistan

**Keywords:** omics, NGS, crops, agriculture, breeding

## Abstract

The deployment of omics technologies has obtained an incredible boost over the past few decades with the advances in next-generation sequencing (NGS) technologies, innovative bioinformatics tools, and the deluge of available biological information. The major omics technologies in the limelight are genomics, transcriptomics, proteomics, metabolomics, and phenomics. These biotechnological advances have modernized crop breeding and opened new horizons for developing crop varieties with improved traits. The genomes of several crop species are sequenced, and a huge number of genes associated with crucial economic traits have been identified. These identified genes not only provide insights into the understanding of regulatory mechanisms of crop traits but also decipher practical grounds to assist in the molecular breeding of crops. This review discusses the potential of omics technologies for the acquisition of biological information and mining of the genes associated with important agronomic traits in important food and fiber crops, such as wheat, rice, maize, potato, tomato, cassava, and cotton. Different functional genomics approaches for the validation of these important genes are also highlighted. Furthermore, a list of genes discovered by employing omics approaches is being represented as potential targets for genetic modifications by the latest genome engineering methods for the development of climate-resilient crops that would in turn provide great impetus to secure global food security.

## Introduction

The majority of efforts to increase crop productivity have focused on conventional breeding techniques, such as phenotyping-based selection. The advancement in genomics over the past 20 years has further boosted the precision and efficiency of breeding programs (Varshney et al., 2005) in many temperate crop species (Eathington et al., 2007). Moreover, the scientific community has invested in the development of genomic resources as well as in intelligent decision support systems (a decision support system that makes extensive use of artificial intelligence (AI)) that result in the reduction of the genotype-phenotype gap and provide effective strategies to develop next-generation climate-resilient crop species (Batley and Edwards, 2016). Being sessile, plants are prone to several stresses that limit their yield. A sound technical knowledge of the gene networks that govern plant stress responses is required to efficiently produce climate-resilient crops. Integrated omics approaches are of great importance as they help in elucidating the essential genetic basis of gene networks that are involved in crop development and plant stress responses (Großkinsky et al., 2018; Muthamilarasan et al., 2019; Naqvi et al., 2022). Omics technologies have been widely utilized to identify the mechanisms involved in plant development, stress responses, yield, and other economically vital traits in important food and fiber crops, such as wheat, rice, maize, potato, tomato, cassava, cotton, etc. In this review, we highlight certain omics-based approaches and their implementation from the perspective of crop improvement. Furthermore, we also described the recent discoveries of crop genomics, transcriptomics, and phenomics and the genes identified through these approaches. Moreover, we have highlighted other technologies (e.g., metabolomics and ionomics) that, if integrated with transcriptomics, can provide deeper insights into the mining of hub genes, which could be employed for developing climate-smart crops. We also provided a list of genes identified from transcriptomics analysis of important food and fiber crops. Lastly, the genes identified from these omics approaches could further be validated through functional genomics techniques, e.g., overexpression, virus-induced gene-silencing (VIGS), and genome editing.

## Genomics-assisted breeding for sustainable agriculture

Several types of molecular markers have been employed for crop improvement. Marker-assisted selection using molecular markers greatly increases the speed of crop breeding by allowing traits to be selected without the need to perform phenotyping. The reduced cost, high read accuracy, and long reads of modern sequencing platforms have further enhanced the application of these molecular markers for crop breeding (Kang et al., 2016). For designing tailored crops, one or more of the following genomics-assisted breeding (GAB) approaches, namely marker-assisted recurrent selection (MARS), marker-assisted backcrossing (MABC), advanced backcross quantitative trait loci (AB-QTL), marker-assisted selection (MAS), promotion/removal of allele through genome editing (PAGE/RAGE), haplotype-based breeding, and genomics selection (GS), have been utilized in breeding programs. The initial step for MAS is the identification of specific molecular markers, which are strongly linked with the genomic regions/QTLs regulating the traits of interest. Ultimately, these individual or multiple QTLs can be pyramided through breeding into an elite cultivar through MABC. Successful stories of MABC include the introgression of *QTL-hotspot* into elite varieties of chickpeas for improved yield under drought conditions (Bharadwaj et al., 2021) and improving the yield and stress tolerance in rice variety IR64. This rice variety has improved cooking quality, earliness, high yield, and disease resistance, which has made it registered worldwide (Swamy et al., 2013; Kumar et al., 2014). Other crops such as barley, sorghum, rice, etc. have also been improved for multiple yield and stress-related traits using a similar approach (Hasan et al., 2015; Gorthy et al., 2017; Xu et al., 2018; Cobb et al., 2019; Kim et al., 2021). MAS has also been applied to improve drought tolerance in multiple crops such as maize, rice, sorghum, wheat, sunflower, and soybean (Borrell et al., 2014; Rama Reddy et al., 2014; Khan et al., 2016). Most agronomically valuable genes were cloned by QTL mapping in plants, i.e., by using biparental mapping populations including doubled-haploid libraries (DHLs), recombinant inbred line (RILs), backcross inbred lines (BILs), chromosomal segment substitution lines (CSSLs), fine mapping, and gene validation by using transgenic approaches. Some valuable genes were also cloned by reverse genetics by using insertional mutant pools (Krishnan et al., 2009; Viana et al., 2019).

The GS approach has gained much attention as it enables the selection of traits based on a larger set of markers rather than a few, as in MAS. The examples exhibiting the potential application of GS in cereal breeding included the transfer of eyespot (*Rhizoctonia cerealis*) resistance genes, *Pch1* and *mlo*, for barley powdery mildew, and recessive resistance genes *rym4/rym5* against barley yellow mosaic viruses (Varshney et al., 2021). The evaluation of GS mainly depends on the genomic-estimated breeding values (GEBVs), and to calculate GEBVs, intensive phenotypic and genome-wide marker information is utilized. The benefit of GEBVs is that they allow the prediction of better-performing individuals compared to their parents and are fit for the next breeding cycle; they can also enter directly into the pipeline for variety release (Crossa et al., 2017). The breakthrough success stories in which GS applied for cultivar improvement against diseases include blast in rice, rust in wheat, and bacterial blight (Viana et al., 2019). Moreover, among abiotic stresses, tolerance to salinity, submergence, and drought remained the preferred traits for improvement. Knowledge of specific marker-trait associations is not required for GS. However, the inclusion of a substantial set of markers, such as outcomes of genome-wide association studies (GWAS), into GS models has improved the prediction accuracy (Li et al., 2018). Thus, GS has attracted attention in plant breeding over traditionally employed strategies. With the availability of effective and economical genotyping platforms and advancements in predictive algorithms, GS is anticipated to be a regular method like MAS/MABC in crop breeding programs. The haplotype-based GWAS and selective sweeps are crucial explanations for understanding genetic diversity in the field of population genetics and genomics, particularly when researching the evolution, adaptability, and stress responses of plant species (Shokat et al., 2020; Bhat et al., 2021; Shokat et al., 2023). A study involving diverse exotics and historical elites developed 2,867 pre-breeding lines for agronomic traits. The study revealed selection footprints and exotic-specific associations, and it uncovered connections specific to invasive species and selection footprints. Many pre-breeding lines contained substantial exotic contributions, despite bias in favor of elite genomes. The selected seven lines were subjected to a varietal release process, and 95 lines have been adopted by national breeding programs for the improvement of the germplasm (Singh et al., 2021). Multiple haplotype and SNP-based model analyses were used to elucidate significant associations within the selection sweeps in tomatoes, which revealed evolutionary insights and potential candidate genes regulating the fruit metabolite content and weight (Zhao et al., 2022). The genomic characterization through NGS and phenotyping data showed 16.1%–25.1% exotic imprints, among which a favorable rare haplotype on chromosome 6D was detected to show minimal grain yield loss upon heat stress. The SNP region annotation showed hits with the isoflavone reductase IRL-like protein of wheat progenitor *Aegilops tauschii*. The overall positive contribution of exotic germplasm was demonstrated, and it was inferred that selected sweeps could be potentially used to secure food insecurity, particularly under climate change threats (Singh et al., 2018).

## Pangenomics: capturing the genetic diversity in a species

Increased genomic sequence information from diverse accessions has allowed the development of pangenomes (Zhou et al., 2015; Varshney et al., 2017). Pangenomics is an ideal and comprehensive approach to capturing all the variations in a species as well as representing the combined genetic repertoire of a species. A pangenome generally consists of two components: the core genome and the dispensable genome. Plant studies have discovered that the core genome has a larger size, contributing the maximum portion of genes (Zhao et al., 2018) while the dispensable genome is more likely to contain polymorphic genes, which could account for survival and adaptation in diverse environments. The comparison of the wild species’ core genome and the dispensable genome of cultivated species uncovers the effect of domestication (Li et al., 2014). At present, pangenomes of several crops, including wheat, rice, soybean, sesame, and tomato, have been published, revealing structural variations and eliminating the single-sample bias of “reference” genomes. Pangenomics has the capability to exhibit an almost full assessment of the diversification existing in a plant species (Montenegro et al., 2017; Yu J. et al., 2019). Recently, a tomato pangenome has been assembled from 725 phylogenetically and geographically distinct accessions. The recognition of 351 Mbp of sequences that were missing in the reference genome was done using a map-to-pan strategy, which also detected a 4-bp substitution in the *TomLoxC* gene’s regulatory region entailing their role in modification in fruit flavor, thus highlighting the selection of fruit quality during the course of domestication (Gao et al., 2019). The advent of robust long-read sequencing technologies and bioinformatics tools is making pangenomics more powerful to aid in discovering crucial genes for trait improvement in major crops.

## Exome sequencing applications in crop improvement

Exome sequencing enables researchers to pinpoint important genes involved in the improvement of traits like disease resistance, heat tolerance, and drought resistance by staying focused on the protein-coding portions of the genome. Exome sequencing is utilized for capturing and sequencing 1%–2% of high-value genomic regions, enriched for functional variants and low repetitive regions. It has proven successful in solving biological questions, understanding molecular variation, marker development, and developing genomic resources for complex crop plants (Kaur and Gaikwad, 2017; Bayer et al., 2019; Xiong et al., 2023). Exome-capturing sequencing yielded 27.8 Gb data, identifying 217,948 SNP and 13,554 Indels in wheat, where functionally important SNPs and Indels were identified at 5.0% and 5.3%, respectively. The exome variations in 12 mutant wheat lines provided insights into mutagenic effects, and functionally enriched genes were found in metabolic pathways like plant–pathogen interactions and ADP binding (Li et al., 2022). The G1674A mutation in a barley gene on chromosome 1HL, encoding cellulose synthase-like C1 protein (HvCSLC1), was identified through whole exome sequencing. It was inferred that this mutation leads to the retention of the second intron and premature termination of the HvCSLC1 protein (Gajek et al., 2021). The combined bulk segregant analysis and whole exome-capturing methods employed in potatoes for studying tuber sprout elongation corroborated different QTL sites, helped to narrow down the related genomic regions, and discovered novel QTLs (Sharma et al., 2021). Overall, with this focused strategy, crop development efforts are more precisely made while simultaneously speeding up the breeding process. Exome sequencing, along with other omics technologies, provides breeders with insights that allow them to develop food and fiber crops that can survive in changing environmental conditions, which eventually contributes to a more sustainable and resilient global food supply.

## Transcriptomics as a tool to discover vital genes

Transcriptomics aids in investigating the differential gene expression and identification of potential genes involved in response to a particular biotic or abiotic stress. Identification of important genes and elucidation of gene expression is thus a potent strategy to develop crops with improved traits (Abdurakhmonov et al., 2016). The availability of well-annotated reference genomes through NGS in the postgenomic era has enabled robust transcriptome profiling. RNA-sequencing (RNA-seq) provides a global representation and coverage of differential gene expression, along with the detection of novel transcripts. Several transcriptome studies have shed light on gene and transcript profiling in crop plants. NGS-based transcriptomics has been utilized for all types of RNA with the advances in massively parallel sequencing platforms. NGS-based RNA sequencing techniques include RNA-seq (whole transcriptome quantification or assembly), small RNA-seq (characterization of small RNA, including micro- and noncoding RNA), PRO-seq (detection of nascent RNA), degradome-seq (typically for miRNA target prediction), SMART-seq (quantification of low input RNA), and ScRNA-seq (detection of gene expression in an individual cell) (Dong and Chen, 2013; Olsen and Baryawno, 2018). The latest bioinformatics tools also provide help in the identification of hub genes through weighted co-expression analysis and genome-wide analysis of gene families (Zaidi et al., 2020; Ehsan et al., 2023). Alternative splicing studies through transcriptomics allow the investigation of genetic diversity in different crops (Glushkevich et al., 2022; Farooq et al., 2023). The innovations in NGS technology have empowered gene expression profiling and annotation of transcriptomes in major food and feed crops, including wheat, rice, maize, potato, tomato, cotton, and cassava, under different conditions and stimuli. The important genes identified in recent years by RNA-seq-based transcriptomics linked to certain responses in major crops are highlighted in **Table 1**.

**Table 1:** Potential gene targets identified via transcriptomics in food and fiber crops.

Variety	Condition or stress	Tissue	Sequencing platform	Approach	No. of DEGs or variants	Important genes/pathways	Reference
Wheat							
Nongda 015 and FZ30	Powdery mildew	Leaf	Illumina HiSeq 4000	2-step bulked segregant RNA sequencing (BSR-Seq)	31 and 20	*Pm5e*	(Xie et al., 2020)
Yunong211	Dithiothreitol and tauroursodeoxycholic acid for endoplasmic reticulum stress	Seedling	Illumina HiSeq	RNA-Seq	8,204	Photosynthesis-related genes, antioxidants, phytohormones, transcription factors	(Yu X. et al., 2019)
PBW677 and PBW703	Nitrogen use efficiency	Root and shoot	Illumina Nextseq500	RNA-Seq	2,406	ABC and SWEET transporters, MYB, bHLH, WRKY, zinc-finger nuclease	(Kaur et al., 2022)
Zhengmai 366 and Chuanmai 42	Drought	Root	Illumina HiSeq 6000	RNA-Seq	11,083	16 dehydrin genes	(Xi et al., 2023)
Rice							
IR36 and Weigu	Salinity	Bursting bud	Illumina HiSeq X Ten	RNA-Seq and QTL-Seq	5	*OsSAP16*	(Lei et al., 2020)
Sahabhagidhan and Geetanjali	Cold	Leaf	Illumina HiSeq2000	RNA-Seq	13,930 and 10,599	AP2/ERF, MYB, WRKY	(Pradhan et al., 2019)
02428 and YZX	Seed vigor	Seed and seedling	Illumina HiSeq	GWAS, QTL, and RNA-Seq	44	*OsEXPA17*, *OsLEA4*, *hsp20*, *OsGH3.8*, GA, and IAA-responsive genes	(Guo T. et al., 2019)
IR64 and Apo	Drought	Leaf	Illumina GAIIx	RNA-Seq	170 and 4	Dehydrin, MYB, NAC, zinc finger, bZIP, HSF-type DNA-binding protein	(Ereful et al., 2020)
Maize							
Zao 8-3 and Ji 853	Low temperature	Seed embryo	Illumina NovaSeq 6000	GWAS and RNA-Seq	10	MAPK and fatty acid metabolism	(Zhang et al., 2020)
B73	Nitrogen stress	Seedling	Illumina HiSeq 2500	Small RNA-Seq	226	miR169, miR398, miR408, miR1214, miR2199	(Yang et al., 2019)
K12 and W64A	Deep seeding	Mesocotyl	Illumina NovaSeq	BSA-Seq and RNA-Seq	24	Cell wall, phytohormones, circadian clock-related genes	(Zhao and Niu, 2022)
Potato							
Kufri Gaurav	Nitrogen use efficiency	Leaf, root, and stolon	Ion Proton	RNA-Seq	206, 144, 775	Superoxide dismutase, GDSL esterase lipase, proline-rich proteins, probable phosphatase 2C, nitrate and sugar transporters, SPX domain, VQ motif, bHLH	(Tiwari et al., 2020)
Longshu No. 3	Wound healing	Tuber	Illumina HiSeq 2500	RNA-Seq	7,665	WRKY, NAC, MYB, sugar and starch metabolism, phytohormone regulation	(Jiang et al., 2022)
Vanderplank and Innovator	Powdery scab	Tuber	Illumina HiSeq 2000	RNA-Seq	2,058	StMRNA, StWRKY6, StUDP, StLOX, StSN1, StPRF	(Lekota et al., 2019)
Tomato							
LA1698 and LA2093	Heat	Leaf	BGISEQ-500	RNA-Seq and QTL-Seq	23,458	SlCathB2, SlGST, SlUBC5, and SlARG1	(Wen et al., 2019)
Moneymaker	Short- and long-term hypoxia	Root	Illumina Nextseq500	RNA-Seq	267 and 1,421	CS9, RBOHB, CAT, MT2B, and ACO1	(Safavi-Rizi et al., 2020)
Local variety	Heat	Leaf	Illumina HiSeq 2500	RNA-Seq and proteome analysis	91	HSPs, HSFs, BAGs, NAC, MBF1C	(Ding et al., 2020)
Ailsa Craig and SlBES1-RNAi-8	Fruit softening	Fruit	Illumina Miseq	RNA-Seq	24	SlBES1 and PMEU1	(Liu et al., 2021)
Cassava							
South China 6068	Waterlogging	Leaf and Root	Illumina	RNA-Seq	2,538 and 13,364	MYBs, WRKYs, NACs, AP2/ERFs, glycolysis, photosynthesis, and galactose metabolism	(Cao et al., 2022)
8 cassava varieties	Cassava brown streak disease	Leaf	Illumina HiSeq 2500	RNA-Seq	8,971	Cinnamic acid, PAL1, PAL2, and chalcone synthase	(Kavil et al., 2021)
Arg7 and W14	Abiotic and biotic stresses	Leaf, stem, and root	Illumina GA II	RNA-Seq	91	MePOD genes	(Wu et al., 2019)
Cotton							
*G. hirsutum* acc. TM-1 and *G. barbadense* cv. Hai7124 and acc. 3-79	Fiber development	Buds	Illumina Novaseq	RNA-Seq and co-expression analysis	1,850 and 1,050	GhP2C72, bHLH, MYB, GhIAA16, HD-ZIP, TCP, GhARF2b, WRKY	(Zhang J. et al., 2022)
*G. arboreum* (Ravi)	Whitefly-mediated CLCuD	Leaf	Illumina HiSeq 2500	RNA-Seq and co-expression analysis	50	CRT, β-1,3-glucanase, HSP40, HSP70, NADH, COX1, COX3, MYB, NRT1/PTR family	(Naqvi et al., 2017)
*G. arboreum* (FDH 228)	Drought and whitefly	Leaf	PacBio IsoSeq and Illumina	RNA-Seq	1,343	CRT1, ERF, bZIP, bHLH, ColI, JAZ1, WRKY, MAPK	(Farooq et al., 2023)
*G. hirsutum* (Karishma)	Whitefly-mediated CLCuD	Leaf	Illumina HiSeq 2500	RNA-Seq and co-expression analysis	53	AOS, MYB, NAC, bHLH, Auxin, cytokinin, ABA, ethyltransferases	(Naqvi et al., 2019)
*G. hirsutum* (Mac7)	Whitefly-mediated CLCuD	Leaf	Illumina HiSeq 2500	RNA-Seq and co-expression analysis	55	NRT1/PTR family, nitrate reductase, IAA4, SAUR-36, cytochrome P450, E3 ubiquitin-protein ligase	(Zaidi et al., 2020; Aslam et al., 2022)
*G. hirsutum* SG747 and *G. barbadense* Giza75	Oil accumulation	Ovule	Illumina HiSeq 2500	RNA-Seq and co-expression analysis	14	*GhCYSD1*, *TAG*, *FAD3*, *BGAL*	(Song et al., 2022)

## Metabolomics, ionomics, and proteomics

Metabolites have essential roles in plant growth, development, yield, and defense mechanisms. Metabolite profiling through metabolomics is a vital tool for studying crop interactions with environmental stresses. Different techniques being utilized to study crop metabolites include gas chromatography-mass spectrometry (GC-MS), liquid chromatography-mass spectrometry (LC-MS), and nuclear magnetic resonance (NMR), each with their own sample preparation protocols and sensitivity (Pretorius et al., 2021). Metabolomics predicts the biochemical markers linked to phenotypic traits, enabling it to be used as a primary detection tool for the identification of favorable traits, which in combination with genetic analysis can be exploited in crop breeding programs (Peng et al., 2015; Razzaq et al., 2019; Raza, 2020). Comparative metabolomics in the roots and leaves of soybean cultivars (sensitive vs. moderately tolerant) through NMR exhibited primary and secondary metabolites. Among these metabolites, alanine, acetate, citrate, GABA, sucrose, and succinate were found to accumulate in plant roots under flooding conditions, however low levels of these metabolites were detected in leaves (Coutinho et al., 2018). Whitefly-resistant and susceptible cassava accessions were compared through metabolomics, which showed that low levels of lignification are associated with whitefly susceptibility (Perez-Fons et al., 2019).

Ultra-performance liquid chromatography-mass spectrometry (UPLC-MS) has been utilized to study comprehensive metabolite profiling of drought-tolerant and sensitive genotypes of Chinese wheat. Guo et al. showed that seedlings of drought-tolerant wheat genotype harbored higher levels of phenolics and 13-fold higher thymine than drought-sensitive genotype (Guo et al., 2020). GC-MS analysis was done for fatty acids profiling in cottonseed (Illarionova et al., 2020) and NMR-based metabolomics has been used to explore metabolites in Bt vs. non-Bt cotton for insect resistance (Shami et al., 2023).

The advances in functional genomics, along with the availability of statistical and bioinformatics tools, allow metabolic profiling to be used as a phenotypic input for genetic association studies, like QTL, thus facilitating crop improvement. The metabolome analysis of 81 accessions of barley under drought and heat stress revealed 57 metabolite QTLs, which were mostly involved in antioxidant defense responses (Templer et al., 2017). Metabolite-based GWAS is another powerful tool to link genetic factors with primary and secondary metabolites. It provides a prospect for identifying candidate genes by exploiting the information from integrated genetics and metabolites. This approach was used efficaciously in tomatoes and detected 44 loci associated with fruit metabolites (Sauvage et al., 2014). mGWAS in 175 rice accessions showed 323 associations among SNPs and metabolites (Matsuda et al., 2015). Another mGWAS study displayed 16 metabolites related to threonine-producing genes in rice under abiotic stress (Muthuramalingam et al., 2018). Thus, metabolomics has great potential to identify candidate genes and quantitative loci that can be used for crop improvement.

Ionomics is another powerful approach, introduced around a decade ago which provides information on the metabolism of elemental composition in plants. It is a high-throughput technique to study the organism’s molecular mechanistic basis of mineral nutrients and their trace element components (also termed the ionome) (Huang and Salt, 2016). For instance, the functional analysis of wheat ionome showed variation in sulfur and phosphorous content associated with grain’s phenotype (Fatiukha et al., 2020). Furthermore, the genome-ionome linkage study in rice revealed 12 micronutrients linked to brown rice, which exhibited its nutrient-dense properties (Pasion et al., 2023). Ionome study combined with GWAS and QTL analysis has shown that shoot and root ionomes in rice were associated with 114 genomic regions where the most significant regions were associated with cadmium, manganese, molybdenum, and sulfur, thus displaying the strength of this approach to manipulate and interrogate the complex traits (Cobb et al., 2021). Ionome and transcriptome combined analysis of two cotton varieties under salinity stress showed accumulation variation of different nutrients in different plant tissues and expressional changes in ion transport-related genes (Guo H. et al., 2019).

Proteomics allows for the study of expressed proteins in crops under specific conditions. A combination of crop proteomics with advanced phenomics and other omics technologies can further assist in the breeding of climate-smart crops (Komatsu et al., 2013). Proteomic studies most predominantly use two-dimensional gel electrophoresis (2-DE) and liquid chromatography (LC)-based techniques that bring forth the proteomes as well as post-translational modifications. Proteomic analysis of soybean varieties by a 2-DE-based procedure under drought and heat stress demonstrated 25 important proteins (Das et al., 2016). The combined metabolome and proteome of maize inbred lines and hybrids showed an abundance of photosynthesis-related proteins, depicting the correlation of hybrid vigor with efficient removal of toxic compounds in hybrids through photorespiration and higher levels of photosynthesis (Li et al., 2020). Comparative proteomics in two rice cultivars under H_2_O_2_ stress revealed proteins related to oxidative metabolism, photosynthesis, and cell defense mechanisms (Bhattacharjee et al., 2023). Metabolomics coupled with proteomics in cassava cultivars under Sri Lankan cassava mosaic virus stress linked results from both approaches and identified pathways involved in plant viral interactions (Siriwan et al., 2023). Thus, proteomics can deliver candidate genes that could be utilized for marker-assisted breeding programs (Jan et al., 2023).

## Phenomics, artificial intelligence, and speed breeding

Phenotypic information is crucial to be utilized in crop breeding; however, recording the phenotypic information in breeding programs remains laborious and time-consuming. The advances in high-throughput computing, remote sensing, artificial intelligence, machine learning, and robotics have made automated phenotyping possible through an approach known as phenomics (Ohyanagi et al., 2022). High-throughput phenomics allows for the measurement of different plant traits, including stress and disease, with automation and precision. A phenomics-based collection of large datasets can be handled, analyzed, and interpreted by modern machine learning algorithms to gain useful intuitions and future predictions of incidence. Neural networks, vector machines, and k-nearest neighbors have been employed in maize, soybean, and wheat for the detection and classification of insect pests (Kasinathan et al., 2021). Hyperspectral imaging, nonimaging spectroscopy, and red–green–blue (RGB) imaging based automated techniques have been emphasized as potential methods for real-time differentiation between crops and weeds in the field for timely management of the weeds (Su, 2020). Artificial neural network-based classification was used to detect blast disease in rice plants with 100% accuracy (Ramesh and Vydeki, 2019). Unmanned aerial vehicle (UAV) imaging and support vector machine classification were used for the crop’s texture information for crop monitoring and yield forecasting (Kwak and Park, 2019). Paudel et al. exploited machine learning models on five crops, including barley, potato, sunflower, soft wheat, and sugar beet in the Netherlands, Germany, and France, which provide workflows to forecast crop yields (Paudel et al., 2021). Hitech phenomics is also aiding in identifying nutrient deficiency and water scarcity in crop-cultivated lands (Sahoo et al., 2023). Another innovation of recent years, speed breeding, i.e., attaining multiple crop generations with reduced generation time under controlled conditions, is an influential approach for efficient plant breeding. Speed breeding, along with advanced AI, provides a platform to accelerate plant breeding programs via linking phenomics and genomics, particularly under climate-changing scenario (Rai, 2022). Recent innovations in precision agricultural technologies like remote sensing, the Internet of Things (IoT), and machine learning can help breeders and farmers make informed decisions and optimize their farming practices. These advanced technologies can play a significant role in sustainable agriculture by improving crop yield, reducing resource wastage, and enhancing overall efficiency (Naqvi et al., 2020).

## Functional genomics approaches for tailored crop improvement

Most of the agronomically important traits are of complex inheritance and challenging to improve. In this case, the mutant and variant alleles can be identified by wide-association studies and QTL mapping (as discussed above), which further need to be functionally validated before being utilized in the breeding program. Understanding the molecular, genetic, and functional basis of a particular gene can help breeders and researchers develop climate-resilient, more productive, and stress-tolerant cultivars.

Conventionally, mutagenesis is an important strategy to introduce mutations, which can be used as a tool for gene functional study and to develop genetic variability. Moreover, to evaluate the mutants and understand gene function, either a forward genetics (from phenotype to genotype) or reverse genetics (genotype to phenotype) strategy can be utilized. Eliminating gene expression or disrupting gene structure exhibits morphological changes in phenotype, providing evidence of the relationship between a gene and its biological function. Although the spontaneous mutation rates are very low (approximately 10^−5^ to 10^−8^) in plants, but mutagenesis is not always effective in gene functional analysis of (Varshney et al., 2005) those genes that are only required under specific biotic and abiotic stress; (2) those genes which are involved in growth and development; and (3) redundant genes because losing these gene function may not lead to morphological changes (Jiang and Ramachandran, 2010; Wang et al., 2013; Viana et al., 2019).

Another strategy of functional genomics is insertional mutagenesis, which includes transfer DNA (T-DNA) insertions, retrotransposon, and transposon tagging. These strategies have been widely used in developing rice mutant libraries. In an analysis of 206,668 insertion flanking sequence tags (FSTs), it was found that 32,459 rice genes have already had insertion tags, and about 50% of predicted protein-coding genes have been equipped with insertional mutagenesis. This study showed the importance of insertional mutagenesis but also had some drawbacks, such as manual manipulations and high cost. However, new tools with more directed, gene-specific methods are needed.

Over the past decade, several genes with substantial phenotypic effects have been functionally validated in different crops via clustered regularly interspaced short palindromic repeats (CRISPR)/CRISPR-associated protein 9 (CRISPR/Cas9)-based genome editing (GE) to improve crop performance against changing climatic conditions. A key feature of CRISPR/Cas9 is the generation of double-stranded breaks (DSBs) of DNA at target loci, which can further be repaired by two cellular mechanisms: nonhomologous end joining (NHEJ) and homology-directed repair (HDR). This tool offers to target various sites simultaneously by utilizing multiple sgRNAs while expressing a single Cas9 or Cpf1 protein (Chen et al., 2019).

Crop-specific functional genes have been exploited to generate gene-edited crops, and approximately more than 60 success stories have been published for drought tolerance, better cell-wall expansion, improved oil quality, and other plant traits. Furthermore, the crop genes that have been exploited by the pathogens for virulence and pathogenicity can be targeted through CRISPR/Cas9, providing an opportunity to break the life cycle of the pathogen (Mahmood et al., 2022).

Several CRISPR-Cas nucleases and their engineered variants have been momentously expanded beyond generating double-stranded DNA breaks (Huang and Puchta, 2021). This technology has advanced immensely owing to *Cas* variants and gene editing approaches aided by apt bioinformatics pipelines. For instance, Cas9 and Cas12a systems have recognized different protospacer-adjacent motif (PAM) for the diagnosis of DNA and RNA viruses (Zhu et al., 2020), while the SHERLOCK system has been employed in soybeans for genotyping and quantification of different traits using crude extracts (Abudayyeh et al., 2019).

Through genomics and transcriptomics data, it has now become possible to screen vital genes systematically. This is possible by using silencing tools such as RNA interference (RNAi) and VIGS, which reduce the expression of specific host target genes and accelerate the plant’s functional genomics. As recently reviewed (Lacomme, 2015; Mahmood et al., 2023), many agricultural VIGS vectors derived from both DNA and RNA viruses are presently available for a wide range of plant species to knock out/down gene expression for functional genomics. The innovative virus-induced genome editing (VIGE) approach is an upgrade of VIGS based on a CRISPR system that offers gene editing with higher efficiency without typical laborious transformation protocols (Zhang C. et al., 2022).

## Conclusions and future prospects

**Figure 1:**
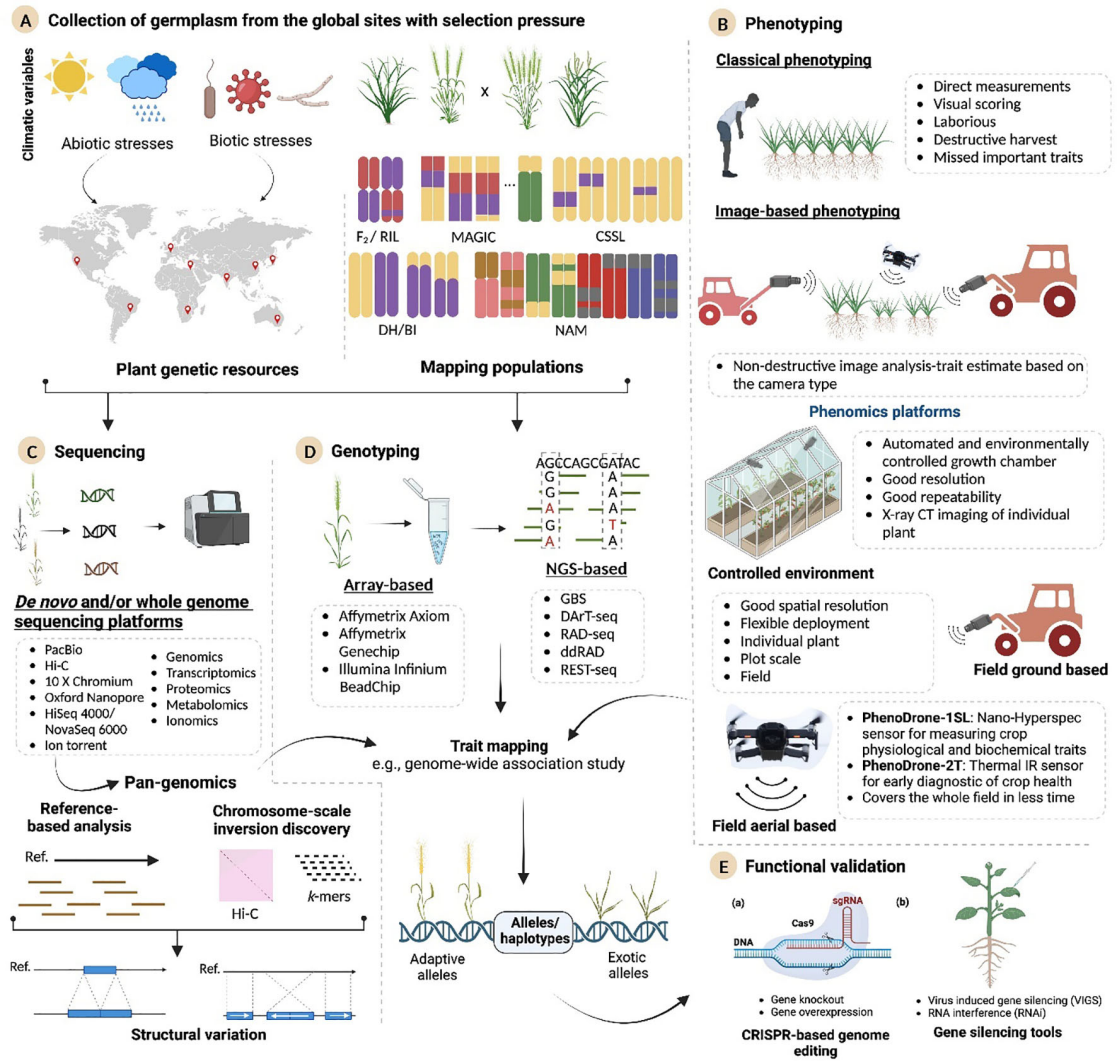
Integrated omics technologies for food and fiber crop improvement. The schematic exhibits a holistic approach that aims to identify the favorable alleles or purge the deleterious alleles in the plant genome for designing climate-resilient crops. **(A)** The plant genetic resources and experimental populations from sites experiencing natural selection pressure are selected to serve as valuable sources for genetic variations. **(B)** Phenotyping approaches consist of classical and high-throughput methods. The advanced imaging platforms span from those operating under controlled conditions to field-based conditions. **(C)** Long-read sequencing methods provide high-quality reference genomes and facilitate pangenomic analysis. **(D)** Advanced high-throughput genotyping approaches develop genome-wide marker information on these panels. **(E)** New genes/haplotypes discovered from analyzing sequence information will be further validated by using the VIGS or CRISPR-Cas system, paving the way forward for enhanced food and fiber crop improvement. MAGIC, multiparent advanced generation intercross; NAM, nested assisted mapping; DHLs, doubled-haploid libraries; RILs, recombinant inbred line; BILs, backcross inbred lines; CSSLs, chromosomal segment substitution lines; VIGS, virus-induced gene silencing; VIGE, virus-induced gene editing; GBS, genotype-by-sequencing; RAD-seq, restriction site-associated DNA sequencing; REST-seq, restriction fragment sequencing.

State-of-the-art sequencing and bioinformatics approaches are being widely used to explore genetic variations in crops. These advances have paved the way for the exploitation of omics technologies such as genomics, pangenomics, transcriptomics, metabolomics, ionomics, proteomics, and phenomics for the identification of potential molecular markers and genes for crop improvement. Functional validation of these genes is possible using VIGS or GE approaches. Identification of genes/markers using integrated omics technologies has the potential to greatly enhance trait selection and, when combined with speed breeding, significantly accelerate crop improvement. In the era of food insecurity and climate change, interconnected utilization of omics technologies, artificial intelligence, speed breeding, and genome editing (**Figure 1**) can certainly revolutionize breeding programs to produce climate-smart food and fiber crops for meeting zero hunger and feeding millions of people across the globe. The unprecedented ability of CRISPR/Cas9 technology has led to the tremendous advances in basic plant research and crop improvement. Certain prospects, such as (1) CRISPR/Cas-mediated multiplex gene regulation as a potential plant synthetic biology tool; (2) exploring cop wild relatives (CWRs) by employing omics technology; (3) improved CRISPR/Cas delivery systems; (4) improved gene editing efficiency by HDR mechanism; and (5) GMO regulatory landscape and concerns, have still been the bottlenecks in the development of climate-resilient and future-smart crops.

## Author contributions

RN: Conceptualization, Data curation, Project administration, Writing – original draft, Writing – review & editing. MM: Data curation, Writing – original draft, Writing – review & editing. SM: Writing – review & editing. IA: Conceptualization, Project administration, Supervision, Writing – review & editing. MA: Conceptualization, Project administration, Supervision, Writing – review & editing.
